# Phase II study of lapatinib in combination with vinorelbine, as first or second-line therapy in women with HER2 overexpressing metastatic breast cancer

**DOI:** 10.1186/2193-1801-3-108

**Published:** 2014-02-22

**Authors:** Helen Kent Chew, Lee Schwartzberg, Suprith Badarinath, Peter Rubin, Grace Shumaker, James Daugherty, Michelle DeSilvio, Janine Mahoney

**Affiliations:** Division of Hematology/Oncology, UC Davis Comprehensive Cancer Center, 4501 X Street, Suite 3016, Sacramento, CA 95817 USA; The West Clinic, Memphis, TN USA; Cancer Specialists of North Florida, Jacksonville, FL USA; Cone Health Cancer Center, Greensboro, NC USA; Jackson Oncology Associates, Jackson, MS USA; Northwest Alabama Cancer Center, Florence, AL USA; GlaxoSmithKline, Collegeville, PA USA

**Keywords:** Metastatic breast cancer, HER2, Lapatinib, Vinorelbine

## Abstract

**Background:**

Lapatinib in combination with capecitabine is approved for the treatment of patients with advanced or metastatic breast cancer whose tumors overexpress the human epidermal growth factor receptor 2 (HER2) and who have received prior therapy including an anthracycline, a taxane, and trastuzumab. Based on our phase I trial, we conducted a single arm, multicenter phase II study of lapatinib in combination with vinorelbine.

**Patient and methods:**

Women with HER2-positive advanced breast cancer, who had received up to one prior regimen for metastatic disease, were eligible. Prior trastuzumab was allowed. Patients received daily lapatinib 1500 mg orally and vinorelbine 20 mg/m^2^ intravenously on days 1, 8 and 15 of a 28-day cycle. The primary endpoint was overall response rate (ORR).

**Results:**

Forty-four patients received the combination treatment, including 48% as second-line therapy. The ORR was 41% (95% confidence interval [CI] 26–55%), including 9% with a complete response. Median progression-free survival was 24.1 weeks (95% CI 17–37 weeks) and median duration of response was 32 weeks (95% CI 18–42 weeks). Nearly 80% of patients did not require a dose reduction in lapatinib, although most patients required one dose reduction of vinorelbine secondary to neutropenia. The most common toxicities were grade 1 and 2 diarrhea, nausea, fatigue and rash, and grade 3 and 4 neutropenia. One case of grade 3 asymptomatic decreased left ventricular ejection fraction event was reported.

**Conclusion:**

The combination of lapatinib and vinorelbine was active, feasible and well tolerated in patients with HER2-positive advanced breast cancer.

**Electronic supplementary material:**

The online version of this article (doi:10.1186/2193-1801-3-108) contains supplementary material, which is available to authorized users.

## Introduction

The addition of the anti-human epidermal growth factor receptor 2 (HER2) monoclonal antibody trastuzumab to chemotherapy improved time to disease progression, objective response rates and survival in patients with metastatic, HER2-positive breast cancer compared to chemotherapy alone (Slamon et al. 2001). This seminal study changed the standard of care for HER2 overexpressed or amplified advanced breast cancer and provided rationale to investigate other targeted therapies in biologically relevant pathways in solid and hematologic malignancies. Trastuzumab has been combined with many other chemotherapeutic regimens (Burstein et al. 2001; Marty et al. 2005; Bartsch et al. 2007; Robert et al. 2006). Despite this progress, most patients will eventually experience disease progression on trastuzumab.

Lapatinib is an oral small molecule tyrosine kinase inhibitor (TKI) that targets the epidermal growth factor receptor (EGFR) and HER2. In combination with capecitabine, a 5-fluorouracil pro-drug, lapatinib improved time to disease progression compared to capecitabine alone in patients with advanced HER2-positive breast cancer that had progressed on trastuzumab (Geyer et al. 2006). This combination was approved by the US Federal Drug Administration (FDA) in 2007.

A phase I study of lapatinib in combination with vinorelbine, a vinca alkaloid microtubule inhibitor, revealed that the combination was feasible with manageable toxicity (Chew et al. 2012). The maximum tolerated dose was vinorelbine 20 mg/m^2^ weekly on days 1, 8 and 15 and lapatinib 1500 mg daily on a 28-day cycle. Dose limiting toxicities included grade 3 infection, febrile neutropenia and diarrhea in this phase I population.

Based on the phase I results, we conducted a multi-institutional phase II trial of lapatinib and vinorelbine in patients with HER2-positive advanced breast cancer who had received up to one prior chemotherapeutic regimen for metastatic disease.

## Patients and methods

This was a single-arm, multi-center, phase II study (ClinicalTrials.gov NCT00709618) evaluating the efficacy and safety of lapatinib plus vinorelbine in women with HER2-overexpressing metastatic breast cancer, who had received no more than one prior chemotherapeutic regimen in the metastatic setting. The study was conducted between June 2008 and May 2012 at 17 centers in the United States.

The study was performed in accordance with the Declaration of Helsinki and approved by the respective institutional review boards of each participating site. For a list of institutional review boards which approved this study, please see additional file 1.

### Patients

Eligible patients were women ≥18 years of age who had histologically confirmed HER2-positive invasive breast cancer (defined as HER2 amplification [>2.2] by fluorescence in situ hybridization or a 3+ positive score by immunohistochemistry) that had progressed after no more than 1 prior chemotherapeutic regimen in the metastatic setting. All prior systemic therapy was completed ≥4 weeks, and all hormonal therapy ≥1 week, before the first dose of study treatment. Patients had stage IV disease at primary diagnosis or at relapse after curative-intent surgery, an Eastern Cooperative Oncology Group (ECOG) performance status of 0–2, an estimated survival of ≥12 weeks, and ≥1 measurable lesion according to Response Evaluation Criteria in Solid Tumours (RECIST) guidelines. Patients were required to have adequate organ and bone marrow function and a cardiac ejection fraction of at least 50% (measured by echocardiogram [ECHO] or multigated acquisition scan [MUGA]). Patients with stable central nervous system metastasis for at least 3 months were permitted. Bisphosphonate therapy for bone metastases was allowed, but treatment must have been initiated prior to the first dose of study medication.

Exclusion criteria included patients with active cardiac, hepatic, or biliary diseases and diseases or surgeries affecting gastrointestinal function. Patients were excluded if they had received prior therapy with lapatinib or vinorelbine (prior trastuzumab was permitted), were undergoing concurrent treatment with anticancer or investigational agents, were pregnant or breastfeeding, or had pre-existing neuropathy of grade 2 or more.

All patients provided voluntary written informed consent in accordance with institutional and federal guidelines.

### Study design

Patients received oral lapatinib, at 1500 mg once daily, plus vinorelbine intravenously, 20 mg/m^2^ on Days 1, 8 and 15, of a 28-day cycle. Granulocyte colony-stimulating factor (GCSF) was not mandated, but could be used in accordance with American Society of Clinical Oncology (ASCO) guidelines (ASCO 2006). Patients continued on study treatment until disease progression or withdrawal from study due to unacceptable toxicity, withdrawal of consent, or lost to follow up.

Efficacy evaluations were performed on all patients at 8-week (±7 days) intervals for the duration of the study, as well as at the end of all study treatment. Safety assessments were performed on all patients at 4-week (±7 days) intervals. Assessments of left ventricular ejection fraction (LVEF) by ECHO or MUGA scan were performed at baseline and q 12 weeks while on study.

### Endpoints

The primary efficacy endpoint was overall response rate (ORR), defined as the percentage of subjects achieving either a confirmed complete response (CR; defined as disappearance of all target lesions) or partial response (PR; defined as at least a 30% decrease in the sum of the longest diameter [LD] of the target lesion, taking the baseline sum LD as a reference), based on confirmed responses from the investigator assessment of best overall response.

Secondary efficacy endpoints included progression-free survival (PFS; defined as the time from the start of treatment until the earliest date of disease progression or death due to any cause, if sooner), overall survival (OS; defined as the time from the start of treatment until death due to any cause) and duration of response (DoR; defined for the subset of subjects who showed a confirmed CR or PR, the time from the first documented evidence of CR or PR until the first documented sign of disease progression or death due to any cause, if sooner). Other secondary endpoints included time to response (TTR; defined for the subset of subjects who showed a confirmed CR or PR, the time from the start of treatment until first documented evidence of partial or complete tumor response) and time to progression (TTP; defined as the time from the start of treatment until the earliest date of disease progression or death due to breast cancer, if sooner). Tumour response and disease progression were based on assessments from the investigator review of radiological scans and medical photographs. RECIST guidelines were used to assess clinical activity and disease status.

Safety assessments included physical exam (vital signs, ECOG performance status), hematology and clinical laboratory evaluations, liver and cardiac toxicity, and monitoring of adverse events (AEs). All grade 4 AEs were defined as serious AEs (SAEs) in this protocol, in addition to those considered life-threatening or resulting in death or hospitalization.

### Statistical analysis

Based on feasibility a total sample size of 60 subjects was planned for enrolment. This study was not powered to provide inference testing for the endpoints.

Efficacy and safety analyses were conducted on the intent-to-treat (ITT) population, comprising all patients who received at least 1 dose of study drug. All subjects’ data were included in analyses up to the time of withdrawal. Subjects who were withdrawn prematurely from the study treatment, but who were not withdrawn from the study at the same time, were included in the analyses regardless of the duration of treatment. Subjects with unknown or missing response were treated as non-responders and included in the denominator for percentage calculation. Exact 95% confidence limits for the tumor response rates were calculated and no statistical comparisons were made in the study.

PFS, OS, DoR, TTR, and TTP were summarized using Kaplan-Meier estimates, along with approximate 95% confidence interval (CI). Greenwood’s formula was used to calculate the standard error of the estimate.

## Results

### Patients

This study was terminated early due to insufficient recruitment. A total of N = 44 patients were enrolled and treated, approximately one-third of the patients (n = 14, 32%) completed the study and 25 (57%) withdrew due to study termination. The most frequent reasons for study treatment discontinuation were disease progression (n = 26, 59%) and decision by subject/proxy (n = 10, 23%).

Patient demographics and disease characteristics are summarized in Table 1. Over one-quarter of the patient population was African or African-American. The median time from initial diagnosis (any stage) was 28.8 months; 48% of the patients had previously received one prior chemotherapeutic regimen in the metastatic setting. Most patients (98%) had received prior anti-cancer treatment, primarily surgery. The most common sites of metastatic disease at baseline were the lungs and lymph nodes, followed by the liver and bone (Additional file 2: Table S1).

**Table 1:** **Patient demographics and disease characteristics**

Characteristic	Lapatinib + vinorelbine (***N***=44)*
**Age, years**	
Median (range)	56.0 (29–82)
**Race,** ***n*** **(%)**	
Caucasian/White	32 (73)
African American/African heritage	12 (27)
**ECOG status at baseline,** ***n*** **(%)**	
0	25 (57)
1	19 (43)
**HER2 status,** ***n*** **(%)**	
FISH+ (with or without IHC+)	27 (61)
IHC 3+ (only)	29 (66)
**ER/PgR status,** ***n*** **(%)**	
ER+ and/or PgR+	23 (52)
ER- and PgR-	21 (48)
**Stage at initial diagnosis,** ***n*** **(%)**	
0	1 (2)
I	3 (7)
II	15 (34)
III	14 (32)
IV	11 (25)
**Number of prior metastatic chemotherapeutic regimen (s),** ***n*** **(%)**	
0	23 (52)
1	21 (48)
**Median time since first diagnosis, months (range)**	28.8 (1–126)
**Prior anti-cancer therapy,** ***n*** **(%)**	43 (98)
Chemotherapy	35 (80)
Immunotherapy	1 (2)
Hormonal therapy	18 (41)
Biologic therapy	35 (80)
Surgery	43 (98)
Radiotherapy	23 (52)

*All patients were female. ECOG, Eastern Cooperative Oncology Group; ER, estrogen receptor; FISH, fluorescence in situ hybridization; HER2, Human epidermal growth factor receptor 2; IHC, immunochemistry; PgR, progesterone receptor.

### Primary efficacy results - overall tumor response rate

The combination of lapatinib and vinorelbine was associated with an investigator-assessed ORR of 41% (n = 18, 95% CI: 26.4, 55.4), with 14 patients (32%) having a PR, 4 patients (9%) a CR and 14 patients (32%) showing stable disease (SD) (Table 2).

**Table 2:** **Efficacy results (ITT population)**

Characteristic	Lapatinib + vinorelbine ( ***N*** =44)
**Investigator assessed Overall Response Rate (ORR)**	
Overall response rate (CR+PR), *n* (%)	18 (41)
(95% CI)	(26.4, 55.4)
**Best response,** ***n*** **(%)**	
Complete response (CR)	4 (9)
Partial response (PR)	14 (32)
Stable disease (SD)	14 (32)
Progressive disease (PD)	5 (11)
Unknown	7 (16)
**Investigator assessed Progression-Free Survival (PFS)**	
**Patients,** ***n*** **(%)**	
Progressed or died due to any cause *	29 (66)
Censored, follow-up ended	15 (34)
**Kaplan-Meier estimate for PFS (weeks)**	
Median (95% CI)	24.1 (16.9, 36.7)
**Time to response (TTR)**	
**Patients,** ***n*** **(%)**	18 (100)
**Kaplan-Meier estimate for TTR (weeks)**	
Median (95% CI)	7.5 (7.1, 8.1)
**Duration of Response (DoR)**	
**Patients,** ***n*** **(%)**	18 (100)
Progressed or died due to any cause	13 (72)
Censored, follow-up ended	5 (28)
**Kaplan-Meier estimate for DoR (weeks)**	
Median (95% CI)	32.0 (18.0, 42.3)

*All deaths were due to breast cancer, therefore progression-free survival and time to progression (TTP) were the same.

CI, confidence interval.

### Secondary efficacy results

The median Kaplan-Meier estimate of investigator-assessed PFS was 24.1 weeks (95% CI 16.9, 36.7) (Table 2). OS was not estimated as the data were not mature and the study had been terminated early with no further data on survival status collected. A total of 14 patients (32%) had died at the end of the study. The median Kaplan-Meier estimate of investigator-assessed TTR and DoR was 7.5 weeks (95% CI 7.1, 8.1) and 32.0 weeks (95% CI 18.0, 42.3), respectively, for patients who responded (CR or PR). The Kaplan-Meier estimate for median TTP was 24.1 weeks (95% CI 16.9, 36.7), which was similar to PFS in this study as all of the PFS events (either progression or death due to any cause) were disease progressions.

### Study treatment exposure

The median duration of exposure to lapatinib was 4.5 months (range, 0–21 months) with a mean daily dose of 1421.9 mg (Additional file 2: Table S2). In total, 59 lapatinib dose interruptions occurred in 17 patients (39%). These interruptions were of short duration (49 interruptions were ≤7 days in duration), and were mainly due to reasons other than hematological toxicities (78%). Twenty-three reductions in lapatinib dose were made in 10 patients (23%); 4%, 30% and 65% of the reductions were due to hematological toxicities, non-hematological toxicities and other reasons, respectively.

In total, 43% of the study population completed at least 6 cycles of treatment with vinorelbine, which was the duration of treatment specified in the protocol. Twenty-five (57%) patients received less than 6 cycles and 12 patients (27%) continued to receive protocol therapy beyond 6 cycles. Thirty reductions in the dose of vinorelbine were made in 26 patients (59%), and were mainly due to hematological toxicities (87%). Twenty five doses of vinorelbine were delayed in 15 patients (34%), and were most commonly due to hematological toxicity (40%) and reasons other than non-hematological toxicity (44%).

### Safety

The most commonly reported AEs were diarrhea, neutropenia, fatigue, nausea, and rash (Table 3). The maximum toxicity grade of the majority of the most commonly reported AEs was grade 2 or 3. The most frequent grade 3 AEs experienced by patients were neutropenia (16%) and diarrhea (11%). A single case of grade 3 asymptomatic decrease in LVEF was reported.

**Table 3:** **AEs by Maximum Toxicity Grade (ITT population)**

Adverse events	Lapatinib + vinorelbine ( ***N*** =44)				
	Number of subjects, ***n*** (%)				
	Total	Grade 1	Grade 2	Grade 3	Grade 4
**Common AEs with >15% incidence**					
Diarrhea	**36 (82)**	22 (50)	9 (20)	5 (11)	0
Neutropenia	**29 (66)**	1 (2)	6 (14)	7 (16)	15 (34)
Nausea	**27 (61)**	17 (39)	8 (18)	2 (5)	0
Fatigue	**23 (52)**	13 (30)	8 (18)	2 (5)	0
Rash	**22 (50)**	12 (27)	9 (20)	1 (2)	0
Constipation	**15 (34)**	10 (23)	5 (11)	0	0
Vomiting	**14 (32)**	8 (18)	5 (11)	1 (2)	0
Hypokalaemia	**12 (27)**	8 (18)	2 (5)	2 (5)	0
Anaemia	**11 (25)**	4 (9)	6 (14)	1 (2)	0
Insomnia	**11 (25)**	7 (16)	4 (9)	0	0
Abdominal pain	**10 (23)**	4 (9)	4 (9)	2 (5)	0
Decreased appetite	**10 (23)**	6 (14)	4 (9)	0	0
Dehydration	**8 (18)**	0	5 (11)	3 (7)	0
Back pain	**8 (18)**	5 (11)	3 (7)	0	0
Dyspepsia	**8 (18)**	6 (14)	2 (5)	0	0
Mucosal inflammation	**7 (16)**	2 (5)	4 (9)	1 (2)	0
Neutrophil count decreased	**7 (16)**	1 (2)	6 (14)	0	0
Pain in extremity	**7 (16)**	4 (9)	3 (7)	0	0
Pyrexia	**7 (16)**	5 (11)	2 (5)	0	0
Cough	**7 (16)**	6 (14)	1 (2)	0	0
**AEs of special interest**					
Hepatobiliary events	**5 (11)**	3 (7)	1 (2)	1 (2)	0
Febrile neutropenia*	**3 (7)**	0	0	1 (2)	2 (5)
Interstitial lung disease	**0**	0	0	0	0
Cardiac events	**1 (2)**	0	0	1 (2)	0

AE, adverse event; ITT, intent-to-treat.

*2/3 patients reporting febrile neutropenia are also counted among the 29 patients reporting neutropenia.

A total of 16 patients (36%) experienced grade 4 AEs (neutropenia, febrile neutropenia and hyperglycemia) but only neutropenia and febrile neutropenia were considered treatment-related. AEs of neutropenia reached a maximum toxicity grade 4 for 15 patients (34%) (Table 3). Of the 30 patients that experienced any grade neutropenia/febrile neutropenia, only 12 patients received GCSF support.

All SAEs experienced by patients are presented in Table 4. No fatal SAEs were reported during the study. Two subjects withdrew from study treatment due to AEs (peripheral neuropathy and nausea).

**Table 4:** **Serious adverse events (SAEs)**

	Lapatinib + vinorelbine ( ***N*** =44)
**Subjects with any SAE,** ***n*** **(%)**	22 (50)
**With** ≥**2 (5%) patients,** ***n*** **(%)**	
Neutropenia	15 (34)*
Diarrhea	3 (7)
Febrile neutropenia	3 (7)
Abdominal pain	2 (5)
Dehydration	2 (5)
**Drug-related fatal SAEs,** ***n*** **(%)**	0 (0)

*Grade 4 laboratory abnormalities were protocol defined as SAEs.

## Discussion

In a study population in which 48% of patients were receiving second-line therapy for metastatic, HER2-positive breast cancer, the combination of lapatinib and vinorelbine led to a 41% ORR, including a 9% CR rate. A randomized phase II trial comparing lapatinib and vinorelbine with lapatinib and capecitabine (VITAL study) reported the primary endpoint of PFS of 6.2 months in each arm, similar to the PFS of 24.1 weeks reported in this study (Janni et al. 2012). Both phase II trials had similar patient populations, but different schedules and dosing of lapatinib and vinorelbine. The current results are comparable to ORRs of 22% and 31%, respectively, with the combination of lapatinib and capecitabine in two other trials (Geyer et al. 2006; Verma et al. 2012). In those trials, patients were required to have had prior therapy with trastuzumab and anthracyclines and/or taxanes (in the adjuvant or metastatic setting). Furthermore, in a more heavily pretreated population that had progressed on trastuzumab, the combination of lapatinib and trastuzumab was associated with a survival benefit over lapatinib alone and an ORR of 10% (Blackwell et al. 2010).

In the current trial, grade 4 toxicities included neutropenia (34%), febrile neutropenia (7%) and hyperglycemia (2%). The most common grade 3 toxicities included neutropenia (16%), diarrhea (11%), dehydration (7%), and nausea, fatigue, rash, hypokalemia and abdominal pain (each 5%). There was one case of grade 3 asymptomatic decrease in LVEF. Similar incidence of grades 3 and 4 neutropenia were reported in the VITAL trial with 15% of participants discontinuing treatment (Janni et al. 2012). Nearly 80% of patients did not require a dose reduction of lapatinib in the current trial. Of the patients who had a dose reduction, the majority (18 of 23) had only one reduction. A greater percentage of patients (59%) required dose reduction of vinorelbine, due mainly to neutropenia and associated with dose delays. Of the 30 patients with any grade neutropenia/febrile neutropenia, only 12 patients received GCSF support. These results suggest that the combination of lapatinib and vinorelbine is a potential treatment option for patients with HER2-positive advanced breast cancer who have had up to one prior chemotherapeutic regimen for metastatic disease.

At the initiation of this trial, lapatinib was the only therapeutic option for HER2-positive disease after progression on trastuzumab. Continued HER2-directed therapy, with either trastuzumab or lapatinib, despite progression on trastuzumab, was associated with improvements in time to disease progression compared to chemotherapy alone in two phase III trials (Geyer et al. 2006; von Minckwitz et al. 2009). The activity in this phase II trial, similar to the VITAL trial, provides patients with additional combination options with lapatinib. Since the start of this trial, other lapatinib combinations have been reported. Lapatinib and paclitaxel improved time to progression compared to paclitaxel alone in a subset of patient with metastatic HER2-positive breast cancer (Di Leo et al. 2008). Likewise, lapatinib and the aromatase inhibitor letrozole improved progression-free survival in postmenopausal women with hormone receptor positive and HER2-positive metastatic breast cancer compared to letrozole alone (Johnston et al. 2009). This endocrine therapy and lapatinib combination was approved by the FDA in 2010.

Since the completion of this trial, other therapies for HER2-positive breast cancer have been approved. Pertuzumab, a monoclonal antibody that binds to a region of the HER2 protein distinct from trastuzumab, has been approved as first-line therapy for metastatic HER2-positive disease in combination with trastuzumab and docetaxel (Baselga et al. 2012) and as neoadjuvant treatment with a similar regimen (Gianni et al. 2012). The drug antibody conjugate, TDM-1, has been approved for patients who have previously received trastuzumab (Verma et al. 2012).

The therapeutic landscape for HER2-positive metastatic breast cancer has advanced in the last decade to include three additional FDA-approved agents beyond trastuzumab. The optimal treatment sequence, the roles of these agents in the adjuvant and neoadjuvant settings, and the significance of trastuzumab resistance will be explored in ongoing and future clinical trials.

## Electronic supplementary material

Additional file 1: **List of IRBs.** (DOCX 14 KB)

Additional file 2: Table S1: Disease burden at baseline. **Table S2.** Study drug exposure. (DOC 34 KB)
