# Supplementary material for: Phase II study of lapatinib in combination with vinorelbine, as first or second-line therapy in women with HER2 overexpressing metastatic breast cancer
Source: Springerplus. 2014 Feb 22;3:108. doi: 10.1186/2193-1801-3-108 (PMC4447850; doi:10.1186/2193-1801-3-108)
Supplement: Supplementary file 1 — Additional file 1: List of IRBs. (DOCX 14 KB) [file 40064_2014_1545_MOESM1_ESM.docx]

**IRBs**
-Quorum Review Institutional Review Board, 1601 Fifth Avenue, Suite 1000 Seattle, Washington, 98101, United States
-Mercy Institutional Review Board, Cancer Resource Center, 4300 West Memorial Road, Oklahoma City, Oklahoma, 73120, United States
-University of California Davis, Institutional Review Board, 2921 Stockton Boulevard, Suite 1400, Room 1429 Sacramento, California, 95817, United States
-Institutional Review Board, Weill Cornell Medical College, 407 East 61st Street, RR110, New York, 10065, United States
-Institutional Review Board, University of Pennsylvania, 3624 Market Street, Suite 3015, Philadelphia, Pennsylvania, 19104, United States
-Western Institutional Review Board, 3535 Seventh Avenue South West, Olympia, Washington, 98502, United States
-Nebraska Methodist Hospital Institutional Review Board, 8303 Dodge Street, Omaha, Nebraska, 68114, United States
-Oregon Health and Science University Research Integrity Office, 3181 Southwest Sam Jackson Park Road, L106 RI, Portland, Oregon, 97239-3098, United States
-Biomedical Research Institute of America, 2525 Camino del Rio South, Suite 300, San Diego, California, 92108, United States
