# Supplementary material for: Phase II study of lapatinib in combination with vinorelbine, as first or second-line therapy in women with HER2 overexpressing metastatic breast cancer
Source: Springerplus. 2014 Feb 22;3:108. doi: 10.1186/2193-1801-3-108 (PMC4447850; doi:10.1186/2193-1801-3-108)
Supplement: Supplementary file 2 — Additional file 2: Table S1: Disease burden at baseline. Table S2. Study drug exposure. (DOC 34 KB) [file 40064_2014_1545_MOESM2_ESM.doc]

**Supplementary Table 1. Disease burden at baseline**

|  | **Lapatinib + vinorelbine(n=44)** |
| --- | --- |
| **Number of organs involved, n (%)**  1  2  ≥3 | 10 (23)  17 (39)  17 (39) |
| **Frequent sites of disease, n (%)**  Lung  Lymph nodes  Liver  Bone  Breast (de novo Stage IV)  Pleura | 25 (57)  24 (55)  18 (41)  12 (27)  7 (16)  7 (16) |

**Supplementary Table 2: Study drug exposure**

|  | **Lapatinib**  **1500 mg daily** | **Vinorelbine**  **20 mg/m^2^** |
| --- | --- | --- |
| **Daily dose (mg), mean (SD)** | 1421.9 (138.3) |  |
| **Number of cycles, mean (SD)** |  | 5.8 (4.7) |
| **Patients with dose reductions, *n* (%)**  **Number of dose reductions, *n* (%)**  0  1  2  3 or more | 10 (23)  34 (77)  8 (18)  0  2 (5) | 26 (59)  18 (41)  22(50)  4 (9)  0 |
| **Patients with dose interruptions, *n* (%)**  **Number of dose interruptions, *n* (%)**  0  1  2  3 or more | 17 (39)  27 (61)  9 (20)  3 (7)  5 (11) |  |
| **Patients with dose delays**  **Number of dose delays, *n* (%)**  0  1  2  3 or more |  | 15 (34)  29 (66)  9 (20)  3 (7)  3 (7) |
